# Identification and Clinical Validation of Key Extracellular Proteins as the Potential Biomarkers in Relapsing-Remitting Multiple Sclerosis

**DOI:** 10.3389/fimmu.2021.753929

**Published:** 2021-12-07

**Authors:** Meng Li, Hongping Chen, Pengqi Yin, Jihe Song, Fangchao Jiang, Zhanbin Tang, Xuehui Fan, Chen Xu, Yingju Wang, Yang Xue, Baichao Han, Haining Wang, Guozhong Li, Di Zhong

**Affiliations:** Department of Neurology, First Affiliated Hospital, Harbin Medical University, Harbin, China

**Keywords:** relapsing-remitting multiple sclerosis, bioinformatics analysis, extracellular protein, protein-protein interactions, biomarkers

## Abstract

**Background:**

Multiple sclerosis (MS) is a demyelinating disease of the central nervous system (CNS) mediated by autoimmunity. No objective clinical indicators are available for the diagnosis and prognosis of MS. Extracellular proteins are most glycosylated and likely to enter into the body fluid to serve as potential biomarkers. Our work will contribute to the in-depth study of the functions of extracellular proteins and the discovery of disease biomarkers.

**Methods:**

MS expression profiling data of the human brain was downloaded from the Gene Expression Omnibus (GEO). Extracellular protein-differentially expressed genes (EP-DEGs) were screened by protein annotation databases. GO and KEGG were used to analyze the function and pathway of EP-DEGs. STRING, Cytoscape, MCODE and Cytohubba were used to construct a protein-protein interaction (PPI) network and screen key EP-DEGs. Key EP-DEGs levels were detected in the CSF of MS patients. ROC curve and survival analysis were used to evaluate the diagnostic and prognostic ability of key EP-DEGs.

**Results:**

We screened 133 EP-DEGs from DEGs. EP-DEGs were enriched in the collagen-containing extracellular matrix, signaling receptor activator activity, immune-related pathways, and PI3K-Akt signaling pathway. The PPI network of EP-DEGs had 85 nodes and 185 edges. We identified 4 key extracellular proteins IL17A, IL2, CD44, IGF1, and 16 extracellular proteins that interacted with IL17A. We clinically verified that IL17A levels decreased, but Del-1 and resolvinD1 levels increased. The diagnostic accuracy of Del-1 (AUC: 0.947) was superior to that of IgG (AUC: 0.740) with a sensitivity of 82.4% and a specificity of 100%. High Del-1 levels were significantly associated with better relapse-free and progression-free survival.

**Conclusion:**

IL17A, IL2, CD44, and IGF1 may be key extracellular proteins in the pathogenesis of MS. IL17A, Del-1, and resolvinD1 may co-regulate the development of MS and Del-1 is a potential biomarker of MS. We used bioinformatics methods to explore the biomarkers of MS and validated the results in clinical samples. The study provides a theoretical and experimental basis for revealing the pathogenesis of MS and improving the diagnosis and prognosis of MS.

## Introduction

Multiple sclerosis (MS), an autoimmune, chronic inflammatory demyelinating disease of the central nervous system, is the number one cause of neurological disability among adults, affecting women twice to three times as often as men (1–3). It is one of the main disability diseases in the global population (4). The clinical features of MS are dissemination in space and time (5). Relapsing-remitting multiple sclerosis (RRMS) mostly occurs in the early onset of MS patients, accounting for about 85% of all MS types. It is the initial phase of MS, which is characterized by reversible episodes of neurological deficits (known as relapsing) that often last for days or weeks, and can be completely relieved after the acute phase (known as remitting) (6). The pathogenesis of RRMS remains incompletely understood, but both genes and environment are believed to contribute (7). At present, it has been found that RRMS in mainly caused by cellular immunity, humoral immunity, and cellular crosstalk in which a variety of extracellular signaling molecules play an important role. They trigger central nervous system inflammation, making immune cell (mainly T cells, B cells, and macrophages) derived from peripheral blood move to the central nervous system and destruct the oligodendrocytes or neurons, resulting in demyelination and axonal damage eventually At present, it has been found that RRMS is mainly caused by cellular immunity, humoral immunity, and a variety of extracellular signaling molecules (8). They can trigger central nervous system inflammation, make immune cells (mainly T cells, B cells, and macrophages) move to the central nervous system, destruct oligodendrocytes or neurons with microglia, and cause demyelination and axonal damage eventually (3). Disease-modifying therapies have gradually been confirmed clinically to be effective in reducing the relapse of RRMS patients, implying that immune regulation plays an important role in RRMS (9). Up to now, subjected to the atypical clinical presentations, RRMS is still difficult to diagnose and lacks effective therapeutic targets. Therefore, the search for biological markers with high sensitivity and specificity is particularly important for studying disease pathogenesis, improving diagnosis, improving prognosis, and providing patients with more personalized treatment strategies.

In recent years, many studies have found that some molecules in the extracellular environment of CNS, mainly secreted proteins, have immunomodulatory effects (10). They are an essential component of the crosstalk between RRMS immune cells (11). Extracellular proteins can be detected in clinical tissues and some body fluids, they may be potential biomarkers or therapeutic targets of RRMS (12–15).

Bioinformatics analysis of transcriptional profiling from microarray is a new method to explore the pathogenesis of autoimmune disorders, identify disease biomarkers and discover therapeutic targets (16). Several MS transcriptomic profiling studies found that the gene expression profiles in peripheral blood of MS patients changed significantly, suggesting that differentially expressed mRNAs and proteins they encoded may be involved in the pathogenesis of MS (17, 18). Due to the scarcity of brain tissue samples from MS patients, there is not much analysis of brain tissue expression profiles in MS. However, the environment in which the lesions are located can most directly reflect the pathological process of the disease. Therefore, analyzing the brain tissue expression profile of MS patients is particularly important for studying the mechanism and biomarkers of MS.

The Gene Expression Omnibus (GEO) database contains the most disease microarray expression profile data so far (19). In this study, we downloaded the MS human brain tissue microarray gene expression profile (GSE5839) from the GEO database, and used R software to screen out the differentially expressed genes (DEGs) between MS human brain tissue and normal human brain tissue samples. Then we selected the extracellular protein-differentially expressed genes (EP-DEGs) from DEGs. Subsequently,we used Gene Ontology (GO) and Kyoto Encyclopedia of Genes and Genomes (KEGG) databases to perform biological function enrichment and pathway enrichment analysis of EP-DEGs. A protein-protein interaction (PPI) network of EP-DEGs had also been established to screen out functional modules and hub genes and extracellular molecules that interacted with hub genes. Finally, cerebrospinal fluid (CSF) samples of RRMS patients and control were collected to validate the expression of key EP-DEGs and their correlation with clinical data at the protein level. This study aims to identify key extracellular proteins that may be involved in the pathogenesis of RRMS, find biomarkers that can improve the diagnosis and prognosis of RRMS clinically, and explore new therapeutic targets.

## Materials and Methods

### Data Acquisition and Processing

The NCBI GEO database (http://www.ncbi.nlm.nih.gov/geo) is an open-access platform for data. It contains the most microarray chip and high-throughput sequencing gene expression profile data to date (20). We download the pre-processed MS human brain tissue microarray dataset (GSE5839) from GEO, which is established on Affymetrix Human Genome U133A Array (HG-U133A). The dataset contains 3 MS patients and 1 non-neurological control brain tissue sample (21). Patients are included according to 2017 McDonald diagnostic criteria, and the patients have not received immunomodulatory treatment. Exclusion criteria includes other inflammatory diseases or autoimmune disorders. We use the Biobase package to normalize the data. According to the annotation information on the platform, the probes are labeled with gene symbols, and multiple probes corresponding to the same gene are randomly selected to remove duplicates, and then the gene expression matrix is obtained.

### Screening of DEGs and EP-DEGs

The limma package in R is currently a powerful method for analyzing DEGs (22). We used it to screen DEGs between patients and control in the GSE5839 dataset. p<0.05 and | log2 fold change (FC) | ≥ 1 were set as the threshold values of DEG identification. After that, we used the Uniprot database to download the extracellular protein gene list GO:0005576 and the Human Protein Atlas (HPA) protein annotation database to download the extracellular protein gene list (23, 24). The two lists were intersected with DEGs. We take the union of the results obtained by the two methods to screen out EP-DEGs, and then analyzed the differential expression of EP-DEGs between the MS group and the control group.

### Functional Enrichment and Pathway Analysis of EP-DEGs

We used the enrichGO and enrichKEGG functions of the ClusterProfiler package in Bioconductor (http://bioconductor.org/packages/release/bioc/html/clusterProfer.html) to perform GO and KEGG enrichment analysis on EP-DEGs. Using the human genome as a background reference, choosing P<0.05 and count ≥2 as cut-off values, we identified the biological processes (BPs), cellular components (CCs), and molecular functions (MFs) of EP-DEGs. We divided EP-DEGs into up-regulated and down-regulated groups, then performed KEGG pathway enrichment analysis on them respectively. Using P<0.05 and count ≥2 as cut-off values, we identified the EP-DEGs enriched pathways. Finally, we used the dotplot and barplot in ClusterProfiler package to show the results of enrichment analysis.

### Construction of the PPI Network of the EP-DEGs and Gene Expression Analysis

The STRING database (http://string-db.org) was used to assess protein-protein interactions (PPIs) in functional protein association network using core factors as query proteins. A PPI network of EP-DEGs was constructed on STRING11.0 (25). Cytoscape visualization was used, with a confidence score>0.4 and a hiding of the unconnected genes. To screen for interactions supported by published literature, we chose text mining evidence. Molecular Complex Detection (MCODE) was applied on Cytoscape to find functional clusters of genes in the PPI network with a degree cutoff=2, node score cutoff=0.2, k-core=2, and max depth=100 (26). The modules with established scores>5 were screened out. We used the CytoHubba plug-in in Cytoscape to find the Top10 node genes in 10 ways, and took the intersection to screen the hub genes. The MCC method is the most accurate in CytoHubba (27). From the selected hub genes, we selected the highest MCC score down-regulated hub gene IL17A as the key gene, and used CytoHubba to predict the first station gene that interacted with it.

### Study Population and Clinical Data Collection

We collected 51 patients with RRMS in the First Affiliated Hospital of Harbin Medical University (Harbin, China) from January 2018 to December 2019, and 20 patients with primary headache as the controls. The entry criteria of the experimental group were strictly in accordance with the McDonald 2017 diagnostic criteria. The entry criteria of the control group were diagnosed according to the International Classification of Headache Disorders (ICHD3) criteria for primary headache (28). Both groups were excluded from patients with other demyelinating diseases, autoimmune diseases, CNS infectious diseases, infections in the past 30 days, or secondary headache. Patients who received immunomodulatory therapies before baseline sampling were excluded. Clinical data of patients were recorded, including name, gender, age, first symptoms, disease course, attack frequency, CSF oligoclonal bands, CSF IgG content, CSF white blood cell count (WBC) and CSF total protein content, to name but a few. Patients were followed up at admission, discharge, 1, 3, 6 months after discharge, and every 6 months for the next years. Study design and the purpose were explained for the participants and informed consent were obtained. The study was approved by the Ethics Committee of First Affiliated Hospital of Harbin Medical University.

### Clinical Outcomes

Two clinical outcomes were used in this study. 1) Clinical relapse. 2) Disability progression. After 6 months from baseline, an EDSS score increase of ≥1 (if baseline EDSS>0) or 1.5 points (if baseline EDSS=0) was defined as disability progress. A new clinical relapse was defined as patient-reported symptoms or objectively observed signs typical for an acute inflammatory demyelinating event in the central nervous system with a duration of at least 24 h, and within one month, in the absence of fever or infection.

### CSF Collection Analysis

**Figure 1:**
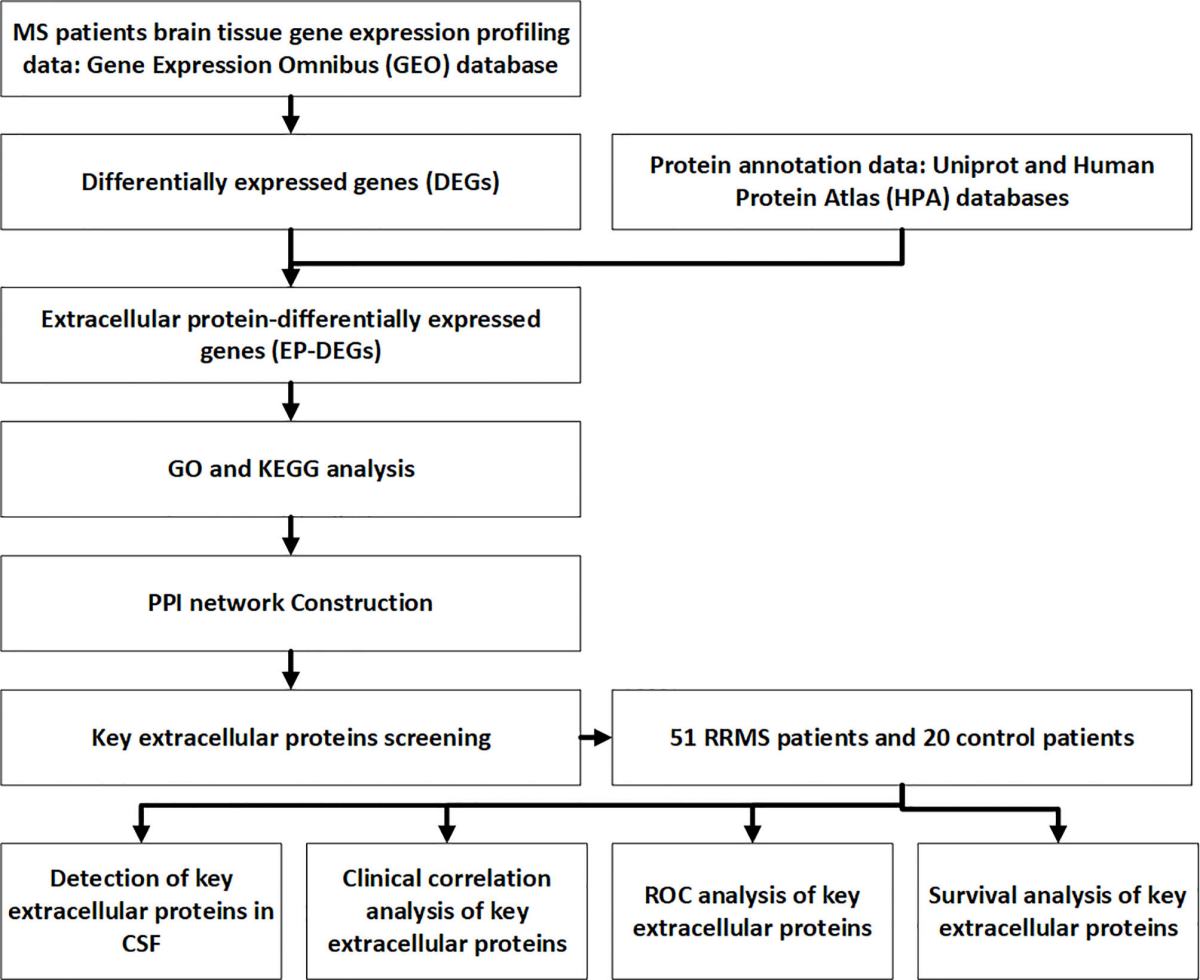
Flow chart of the study.

Cerebrospinal fluid samples obtained by lumbar puncture were collected and stored at -80°C. Samples were immediately transferred into the tubes for ELISA detection. Del-1 (Wuhan Boster), IL17A (Shanghai Enzyme Immunoassay), and resolvinD1 (Shanghai Enzyme Immunoassay) ELISA kits were used to detect the content of Del-1, IL17A, and resolvinD1 in CSF respectively, two technical replicates (wells) for each sample. The protocols of ELISA were performed according to the kit’s instructions: dilute the standard, add 100μl of the sample to the wells of the ELISA kit, incubate the kit at 37°C for 90 minutes. add 100μl of biotin-labeled antibody to the working solution, incubate at 37°C for 60 minutes. Wash the plate, add 100μl of enzyme-labeled reagent, incubate at 37°C for 30 minutes. Wash the plate, add 90mul of color reagent to each well, incubate for 20 minutes at 37°C in the dark. Add 100μl of stop solution to each well to stop the reaction. Measure the OD values at 450 nm by a microplate reader. Calculate the actual content of CSF Del-1, IL17A, and resolvinD1 according to the equation. The flow chart for this study is shown in **Figure 1**.

### Statistical Methods

Biobase package, limma package, ClusterProfiler package, pROC package of R and Cytoscape V3.8.2 software were used to analyze public gene expression data. SPSS V25.0 and GraphPad V8.0.2 software were used for statistical analysis of clinical data. The measurement data are described as mean±SD (normal distribution) or median (P25, P75). Two-tailed independent sample t-test or wilcoxon rank sum test were used to compare the mean difference between the two groups. Correlations were determined by Pearson or Spearman’s analysis. TheAUC was used to evaluate the diagnostic performance of all models, and the differences were compared by the DeLong test. Relapse-free and progression-free survival curves were built using the Log-rank (Mantel-Cox) method. Difference were considered statistically significant at *p<0.05, **p<0.01, ***p<0.001.

## Results

### Identification of DEGs

**Figure 2:**
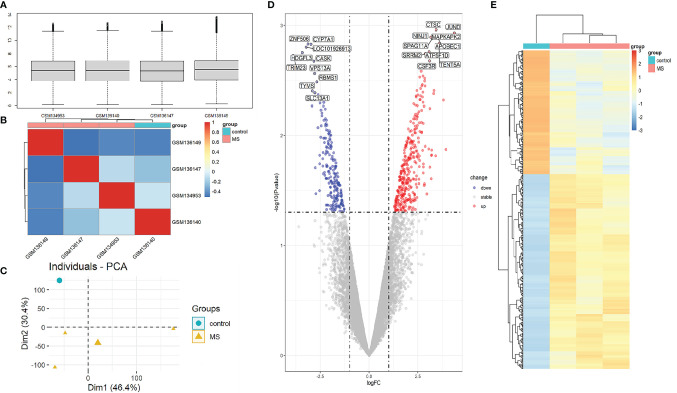
Analysis of gene expression correlation and differential gene expression between MS group and control group in the dataset. **(A)** Boxplot of gene probe expression levels among samples. There was no significant difference in the median and the upper and lower quartile. **(B)** Correlation heatmap between samples. Compared with the control group, the intra-group correlation in the MS group was stronger. **(C)** PCA principal-component analysis. The center points of the MS group and the control group are far apart in space, indicating that the principal components are different. **(D)** Volcano map of all DEGs in the MS group and the control group analyzed by the limma R package. The top 10 up-regulated and down-regulated genes with the smallest P-value are marked on the map. **(E)** Heatmap of all DEGs in MS group and control group.

To compare the difference in gene expression between the MS group and the control group, differential gene expression analysis between samples was performed. The median, upper and lower quartiles, maximum and minimum values of the four sample genes in the GSE5839 data set are basically the same (**Figure 2A**). Correlation analysis showed that the intra-group correlation in the MS group was stronger (**Figure 2B**). Principal-component analysis showed that the center of the MS group and the control group were far apart,indicating that there were differences in gene expression between the MS group and the control group (**Figure 2C**). Differential gene expression analysis set |logFC|≥1 and P<0.05 as DEGs, and a total of 540 DEGs were screened (**Sup 1**). The top three up-regulated genes with the smallest P-value are CTSC, JUND, and NINJ1, and down-regulated genes are ZNF506, CYP7A1, and LOC101926913 (**Figure 2D**). The DEGs heat map showed that DEGs were consistently up-regulated or down-regulated in the MS group, which was significantly different from the control group (**Figure 2E**).

### Screening of EP-DEGs

**Figure 3:**
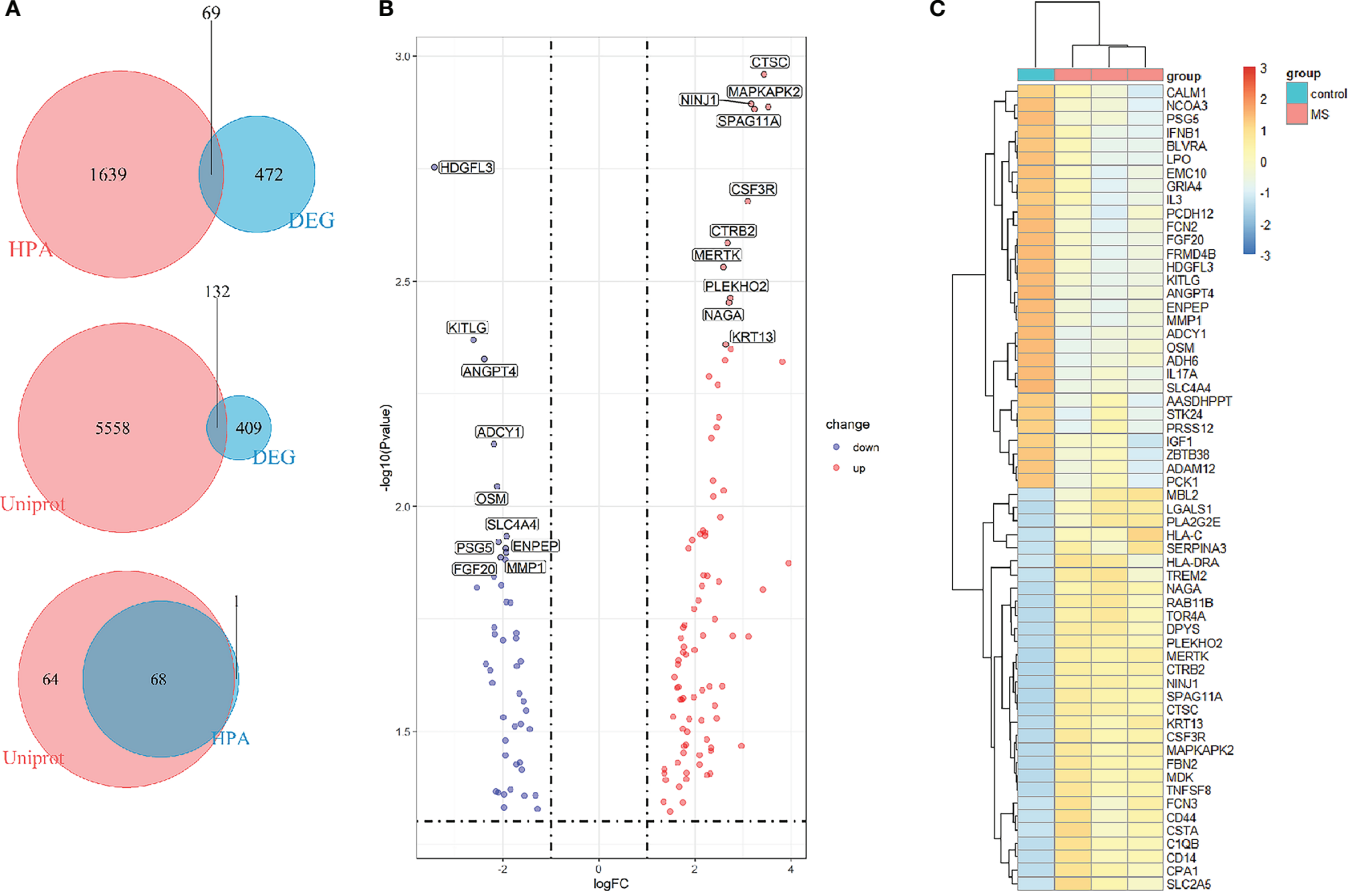
Screening of differentially expressed genes encoding extracellular proteins. **(A)** The genes encoding extracellular proteins annotated in the HPA database were intersected with DEGs, 69 EP-DEGs were screened out. The genes encoding extracellular proteins annotated in the Uniprot database were intersected with DEGs, 132 EP-DEGs were screened out. The genes screened by the two methods were combined to obtain a total of 133 EP-DEGs. **(B)** Volcano map of EP-DEGs in MS group and control group. Mark the top 10 up-regulated and down-regulated genes with the smallest P-value. **(C)** Heatmap of the top 30 up-regulated and down-regulated EP-DEGs.

To find out the genes encoding extracellular proteins that are differentially expressed in the MS group and the control group, we refer to the annotated extracellular protein genes in existing public libraries, screen out EP-DEGs from DEGs and analyze them. The genes encoding extracellular proteins annotated in the HPA database were intersected with DEGs, and 69 EP-DEGs were screened out. The genes encoding extracellular proteins annotated in the Uniprot database were intersected with DEGs, and 132 EP-DEGs were screened out. The EP-DEGs obtained by these two methods were unionized, and only one gene was not overlapped. A total of 133 EP-DEGs were screened, 87 up-regulated and 46 down-regulated (**Figure 3A** and **Sup 2**). The top 10 up-regulated genes with the smallest P-value in the MS group and control group were CTSC, NINJ1, MAPKAPK2, SPAG11A, CSF3R, CTRB2, MERTK, PLEKHO2, NAGA and KRT13. The top 10 down-regulated genes were HDGFL3, KITLG, ANGPT4, ADCY1, OSM, SLC4A4, PSG5, ENPEP, MMP1 and FGF20 (**Figure 3B**, **Table 1** and **Sup 3**). The heatmap of EP-DEGs up-regulated and down-regulated in the first 30 with the smallest P-value shows that SLC4A4, IL17A, ADH6, OSM, and ADCY1 are significantly down-regulated in MS and the clustering distance is close (**Figure 3C**).

**Table 1:** Top twenty EP-DEGs in RRMS human brain tissues (GSE5839).

symbol	logFC	AveExpr	P.Value
CTSC	3.433656544	4.538716532	0.001096231
NINJ1	3.164096409	5.651057054	0.001274412
MAPKAPK2	3.51814957	3.486609084	0.001296023
SPAG11A	3.231901028	3.959978671	0.001309249
CSF3R	3.092993263	4.424081607	0.002097986
CTRB2	2.672838909	4.437588589	0.002595307
MERTK	2.591990121	3.791989497	0.002937676
PLEKHO2	2.731264198	6.234314693	0.003439064
NAGA	2.70318136	4.290420426	0.003526288
KRT13	2.633039893	3.353291543	0.004368255
HDGFL3	-3.428978333	2.240764475	0.001763368
KITLG	-2.609629361	1.62774048	0.004262401
ANGPT4	-2.39160971	3.151151163	0.004698345
ADCY1	-2.187618912	3.19217583	0.007254208
OSM	-2.118575941	2.490019385	0.00901236
SLC4A4	-1.920336454	1.924320091	0.011628523
PSG5	-2.090113143	3.110487048	0.011968366
ENPEP	-1.945700755	1.611113762	0.012368029
MMP1	-1.931833585	1.672140212	0.012628526
FGF20	-2.048333614	2.481671697	0.012946904

### GO and KEGG Pathway Enrichment Analysis of EP-DEGs

**Figure 4:**
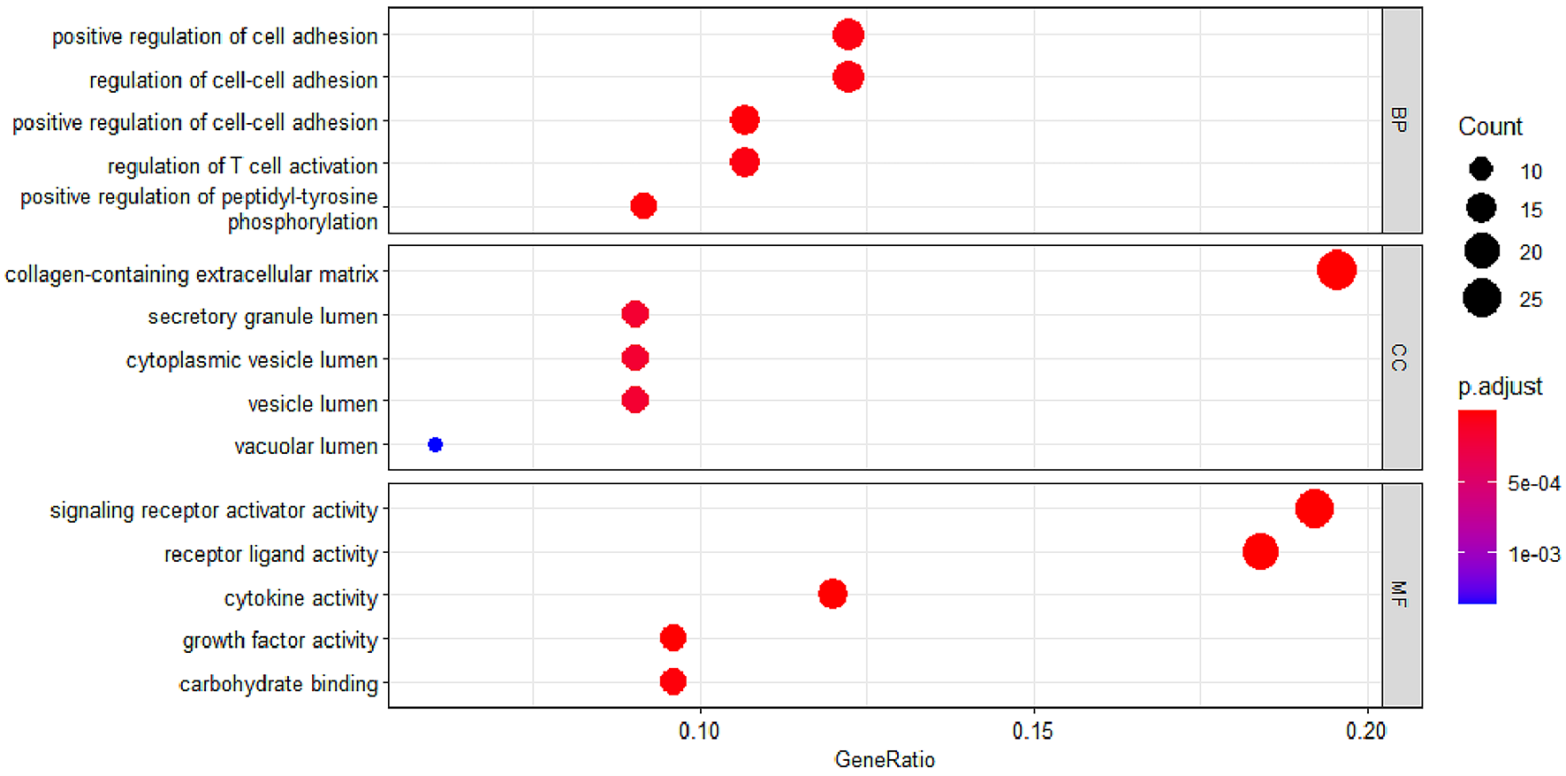
GO enrichment of EP-DEGs. The dotplots show the Top5 processes enriched by EP-DEGs in BPs, CCs and MFs.

**Figure 5:**
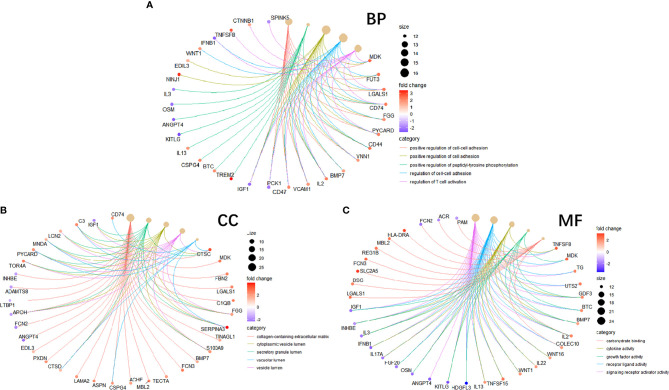
Circle graph in GO enrichment of EP-DEGs. **(A–C)** The circle graph shows the EP-DEGs enriched in the Top5 GO categories of BPs, CCs, and MFs, respectively. The yellow points represent the GO categories, the color of the line delivered by a point indicates the category of the point in the legend, the size of a point indicates the number of the genes it includes.

**Figure 6:**
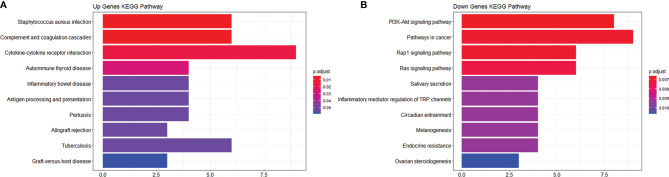
KEGG enrichment analysis of EP-DEGs. **(A, B)** respectively show the pathways to which up-regulated genes and down-regulated genes are enriched.

To study the function of EP-DEGs, we performed GO and KEGG enrichment analysis on EP-DEGs. The enrichGO function in the ClusterProfiler package is used to enrich EP-DEGs from BP, CC and MF respectively. EP-DEGs were mainly enriched in positive regulation of cell adhesion, regulation of cell-cell adhesion of BPs, collagen-containing extracellular matrix of CCs, and signaling receptor activator activity and receptor-ligand activity of MFs (**Figure 4**). The cnetplot function in the ClusterProfiler package is used to display the genes enriched in the top 5 processes with the smallest P-value of BPs, CCs, and MFs. Among them, MDK, LGALS1, CD74, PYCARD, BMP7, IL2, IGF1, IL13, KITLG, ANGPT4, OSM, IL3, EDIL3, TNFSF8 are enriched in at least two aspects of BPs, CCs and MFs (**Figure 5**). With reference to the human genome, the enrichKEGG function in the ClusterProfiler package is used to enrich the up-regulated and down-regulated genes in the KEGG pathway respectively, and the up-regulated genes are enriched in Staphylococcus aureus infection, complement and coagulation cascades, cytokine-cytokine receptor interaction, and autoimmune thyroid disease. Down-regulated genes are enriched in the PI3K-Akt signaling pathway, pathways in cancer, rap1 signaling pathway, and ras signaling pathway (**Figures 6A, B**).

### Establishment of PPI Network and Identification of Hub Genes

**Figure 7:**
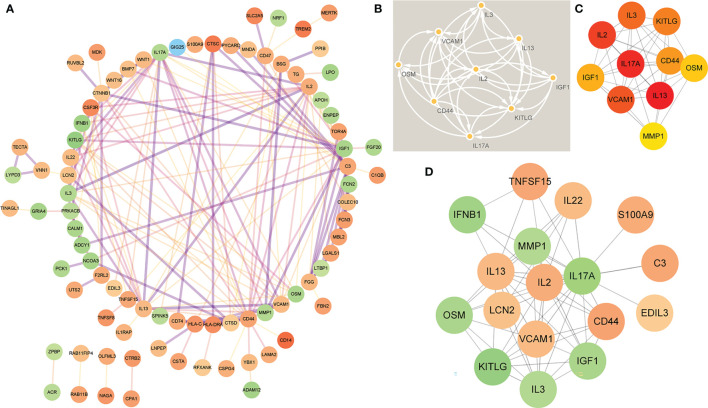
Construction of PPI network of EP-DEGs and screening of hub genes. **(A)** The STRING database is used to construct the PPI network of EP-DEGs, with 85 nodes and 185 edges (the legend is in the **Supplementary Material**). **(B)** The node gene cluster with the highest score constructed by the MCODE plug-in in Cytoscape consists of 9 genes. **(C)** The Cytohubba is used to construct the Top10 hub genes. The figure shows the Top10 hub genes constructed by the MCC method. **(D)** The Cytohubba was used to predict the first stop node genes that interact with IL17A. A total of 16 genes were predicted, 10 up-regulated and 6 down-regulated.

In order to study the interaction between the proteins corresponding to EP-DEGs, the STRING database was used to construct a PPI network of 133 EP-DEGs. Cytoscape software was used to visualize the total PPI network (**Figure 7A**). The PPI network consists of 85 nodes and 185 edges. The darker the color and the wider the edge, the stronger the evidence for the interaction between proteins (see **Supplementary Figure 2** for the legend). The MCODE plug-in in Cytoscape is used to construct functional modules. The results show that there is only one module with an established score>5, consisting of 9 genes and 34 edges (**Figure 7B**). The 10 topological methods of the CytoHubba plug-in in Cytoscape were used to screen top10 hub genes. There are 4 genes in all 10 methods, namely IL17A, IL2, CD44, IGF1 (**Table 2** and **Figure 7C**). IL17A is the gene that exists in the functional module, and the down-regulated gene with the highest score in the MCC method (the most accurate method) (27). CytoHubba was used to construct the first node gene to interact with IL17A. A total of 16 genes were screened out (**Figure 7D**), 10 were up-regulated and 6 were down-regulated.

**Table 2:** Top10 EP-DEGs by 10 topological analysis methods of CytoHubba.

MCC	MNC	Degree	EPC	BottleNeck	EcCentricity	Closeness	Radiality	Betweeness	Stress
IL13	IL2	CD44	IL2	IL2	IL2	CD44	IL2	CD44	CD44
IL17A	CD44	IL2	IL17A	CD44	CD44	IL2	CD44	C3	IL2
IL2	IL13	IGF1	CD44	IGF1	IGF1	IL17A	IL17A	IL2	C3
VCAM1	IL17A	IL13	IL13	C3	C3	IGF1	IGF1	IGF1	IGF1
IL3	VCAM1	IL17A	IGF1	CTNNB1	IL17A	VCAM1	VCAM1	IL17A	IL17A
KITLG	IGF1	C3	VCAM1	IL17A	IL13	IL13	IL13	CTNNB1	CTNNB1
CD44	IL3	VCAM1	IL3	IL13	VCAM1	C3	IL3	CD47	CD47
IGF1	KITLG	CTNNB1	KITLG	CTSD	CSF3R	IL3	C3	LCN2	VCAM1
OSM	OSM	IL3	OSM	CD47	IL3	CTNNB1	KITLG	IL13	LCN2
MMP1	MMP1	KITLG	MMP1	VCAM1	KITLG	KITLG	MMP1	PRKACB	IL3

### Levels of IL17A, Del-1 and ResolvinD1 and Their Correlation With Clinical Data

**Figure 8:**
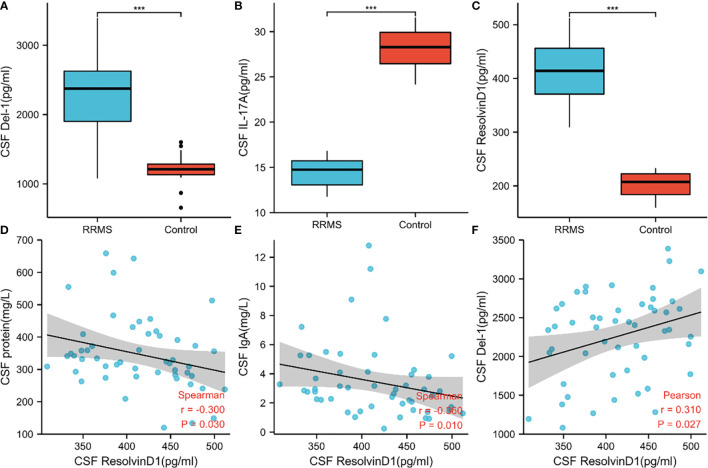
Levels of IL17A, Del-1, and resolvinD1 in CSF of RRMS patients and their correlation with clinical data. **(A–C)** Del-1 and resolvinD1 levels were elevated in RRMS patients, and IL17A level were reduced in RRMS patients. **(D–F)** ResolvinD1 was positively correlated with Del-1, resolvinD1 was negatively correlated with protein and IgA. ***p < 0.001.

In order to verify the key extracellular proteins identified, 51 RRMS patients and 20 patients with primary headache who excluded CNS infectious diseases and recent infections were included (see **Tables 3**, **4** and **Supplementary Material 10** for patient baseline data), and the patients’ CSF was collected to detect IL17A, Del-1 (encoded by the EDIL3 gene) levels. It has been reported in the literature that there is a regulatory relationship between IL17A, Del-1 and resolvinD1, so the level of resolvinD1 in the CSF of RRMS patients was also detected (29). Del-1 and resolvinD1 levels were elevated in RRMS patients, and IL17A levels were reduced in RRMS patients (**Figures 8A–C**). Correlation analysis of the three extracellular molecules and clinical indicators revealed that the level of resolvinD1 was positively correlated with Del-1 in the CSF of RRMS patients, and the level of resolvinD1 was negatively correlated with protein and IgA (**Figures 8D–F**).

**Table 3:** Clinicopathological characteristics of RRMS patients and control samples (N = 71).

Patient demographics and laboratory information	RRMS (N = 51)	Control (N = 20)	P value
Average age	36.12±10.28	37.90±13.19	0.547
Sex (female)	39 (76.47%)	15 (75.00%)	0.238
Oligoclonal band positive	20 (39.2%)	0	0.001**
CSF IgG (mg/L)	32.7 (21.9-54.5)	20.2 (14.85-28.3)	0.002**
CSF IgA (mg/L)	2.85 (1.76-4.14)	2.23 (1.4-2.67)	0.056
CSF IgM (mg/L)	0.45 (0.29-0.89)	0.32 (0.23-0.41)	0.049*
CSF Alb (mg/L)	172 (138-204)	162.5 (124.5-199)	0.494
CSF total cell count (10^6^/L)	5 (0-10)	1 (0-6.5)	0.122
CSF WBC count (10^6^/L)	1 (0-4)	0 (0-1.5)	0.26
CSF protein (mg/L)	328.53 (282.84-403.98)	287.52 (238.04-351.98)	0.089

*p < 0.05, **p < 0.01.

**Table 4:** Baseline characteristics of RRMS patients (N = 51).

Patient baseline characteristics	Enrolled RRMS patients (N = 51)
Frequency of relapses/year	0.91±0.76
Disease duration at admission	2.93±3.52
EDSS score at admission	1.17±0.51
Disease Course/day (from this relapse date to lumber puncture date)	18.49±18.37
**Comorbidities**	
History of hypertension,n (%)	6 (11.76)
History of diabetes,n (%)	0

### Del-1 Diagnostic Efficacy and Survival Analysis

**Figure 9:**
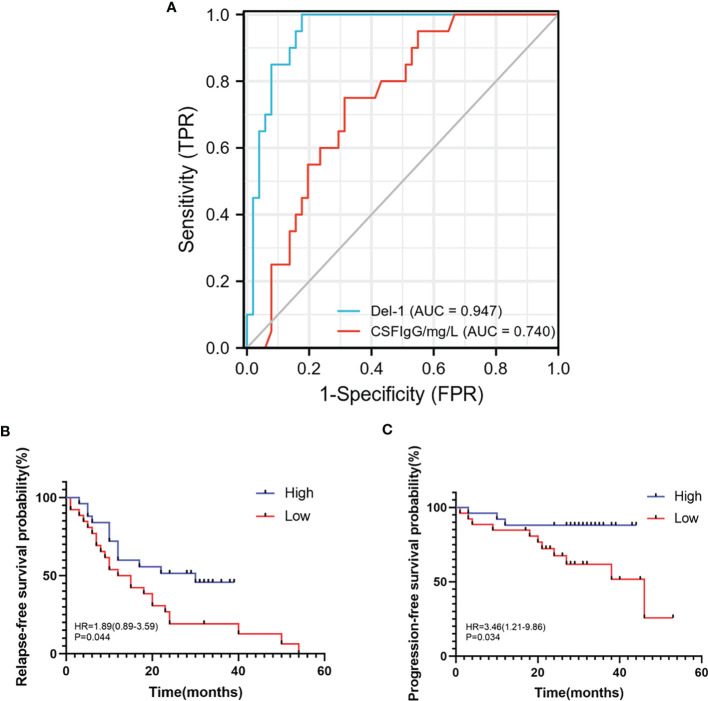
ROC, relapse-free survival, progression-free survival curves of Del-1 in RRMS. **(A)** The area under the ROC curve of Del-1 (AUC=0.947), which is higher than that of IgG (AUC=0.740). **(B)** The median relapse-free survival time was 30 months in the high Del-1 group, the median relapse-free survival time was 13.5 months in the low Del-1 group, the difference was statistically significant (P=0.044). **(C)** The probability of progression-free survival in the high Del-1 group was always higher than 50% during the follow-up period, and the median progression-free survival time in the low Del-1 group was 46 months, the difference was statistically significant (P=0.034).

In order to study the predictive effect of Del-1 on the diagnosis and prognosis of RRMS, ROC curve and survival analysis were performed. Del-1 had high accuracy in the diagnosis of RRMS (AUC = 0.947, 95%CI = 0.898-0.996), and IgG had certain accuracy in the diagnosis of RRMS (AUC = 0.740, 95%CI = 0.623-0.857) (**Figure 9A**), In the diagnostic model of RRMS, the diagnostic efficacy of Del-1 was better than that of IgG, and the result was statistically significant (DeLong’s test, P = 0.002). The cut-off value of Del-1 was 1623.882pg/ml, the Sensitivity% corresponding to this cut-off value was 82.4%, and the Specificity% was 100%. Del-1 was divided into high and low groups according to the median, and survival analysis was performed in RRMS patients (**Figures 9B, C**). The results showed that the median relapse-free survival time in the high Del-1 group was 30 months, and the median relapse-free survival time in the low Del-1 group was 13.5 months, the difference was statistically significant [HR=1.89(0.89-3.59)], P=0.044). The probability of progression-free survival in the high Del-1 group was always higher than 50% during the follow-up period, and the median progression-free survival time in the high Del-1 group was 46 months, the difference was statistically significant [HR=3.46(1.21-9.86)], P=0.034).

## Discussion

### Biological Processes in Which EP-DEGs May Participate in MS

We analyzed the GSE5839 dataset and obtained a total of 541 DEGs. Among the top 3 up-regulated and top 3 down-regulated genes, only 2 genes were marked as extracellular proteins in the Genecards database. Because some nuclear proteins and cytoplasmic proteins cannot be detected in the body fluids of clinical patients, this analysis method did not focus on finding intracellular biomarkers of the disease. We compared DEGs with extracellular protein gene lists in the protein annotation database Uniprot and HPA, and a total of 133 EP-DEGs were screened. We compared the EP-DEGs obtained from the two databases. It was interesting that 68 of the 69 EP-DEGs selected from the HPA database overlapped with Uniprot, and only 1 gene did not overlap, which reflected these two database had good consistency in the annotation of extracellular proteins. GO enrichment showed that EP-DEGs were enriched in collagen-containing extracellular matrix, signaling receptor activator activity, receptor-ligand activity and positive regulation of cell adhension processes. This indicated that extracellular proteins might be mainly secreted into the extracellular matrix by specific cells in the pathological process of MS. They bound to receptors on specific cells as ligands, transmitted signals for cell-to-cell communication, and mediate processes such as cell migration and adhesion. The GO enrichment circle map shows that MDK, LGALS1, CD74, PYCARD, BMP7, IL2, IGF1, IL13, KITLG, ANGPT4, OSM, IL3, EDIL3, and TNFSF8 were enriched in multiple biological processes. These genes might play a more important role in MS. KEGG enrichment analysis showed that up-regulated EP-DEGs were mainly enriched in certain inflammatory pathways, complement pathways and cytokine-cytokine receptor pathways. The result indicated that extracellular proteins in MS might be mainly some cytokines, which mainly activated immunity-related pathways. Down-regulation of EP-DEGs mainly enriched in PI3K-Akt signaling pathway. Studies had found that the PI3K-Akt signaling pathway played an immune-regulatory role in the development of regulatory T cells (30) and the inflammation process of periodontitis (29). An FTY720 analog in the EAE model could inhibit the progression of inflammation by inhibiting the PI3K-Akt signaling pathway (31). These results collectively suggested that the PI3K-Akt signaling pathway might play a regulatory role in the progression of MS inflammation.

### IL17A and EDIL3 May Be the Key Extracellular Proteins in the Pathogenesis of MS

After analyzing the PPI network of EP-DEGs, we found that IL17A, IL2, CD44, and IGF1 were simultaneously present in the Top10 hub genes screened by 10 CytoHubba topology methods and the functional gene modules constructed by MCODE. The involvement of IL17A in the pathological process of MS has been confirmed (32), but the changes of IL17A content in the serum and CSF of MS patients are controversial. Increases in IL17A in the acute phase of MS have commonly been reported (33), but other studies have found no changes or even reductions (13). The results of this study showed that the expression of IL17A was down-regulated, and the verification at the protein level also showed that IL17A was down-regulated. The patients included in this study were not in the active acute phase, so we speculate that IL17A levels may increase in the early stage of MS inflammation, and IL17A levels gradually decrease as the inflammation subsides, but this speculation needs to be further verified.

### Del-1 Could Be Related to the Relapse and Progression of RRMS

The extracellular environment of the brain and spinal cord is CSF. There are a large number of extracellular proteins in CSF that act as messengers to transmit signals (34). Therefore, we detected the levels of IL17A, Del-1 and resolvinD1 in the CSF of MS patients to verify the key extracellular proteins predicted by bioinformatics analysis. The levels of IL17A decreased, the levels of Del-1 and resolvinD1 increased, the trend was consistent with the prediction. Del-1 and resolvinD1 levels were positively correlated, Del-1 and IL17A levels exhibited the opposite trends. The results were consistent with previous literature reports, suggesting that Del-1, IL17A and resolvinD1 might interact with each other to regulate the occurrence and development of MS. Moreover, Del-1 had high accuracy in the diagnosis and prognostic stratification of MS, suggesting that Del-1 might be helpful to the diagnosis of MS. Compared to the high Del-1 group, the median relapse-free survival time and median progression-free survival time in the low Del-1 group was lower, indicating that the baseline Del-1 levels may be related to the relapse and progression of the disease. Whether the patients received immunomodulatory therapies, age, sex, sleep and stress might be the potential confounders in survival analysis. Additionally, no differences in age or sex existed between the groups, moreover, we excluded the patients who had ever received immunomodulatory therapies, which might be the potential confounders in survival analysis, therefore, these aspects may not affect the results. Other aspects are needed to be investigated in detail in the following studies.

### Limitations

This study has some limitations. At present, most of the MS gene expression data in the publically available datasets comes from human peripheral blood, and the human brain tissue gene expression data is relatively few, so the small sample sizes may cause bias in the results. In the future, more MS human brain tissue expression profiles will be needed for further analysis. In the process of screening extracellular proteins, two protein annotation databases were used, but the evidence for protein localization of these annotation databases is mainly derived from published literature. The evidence is not complete, and some extracellular proteins may be missed. We validated the key EP-DEGs at the protein level, not at the transcription level. Because the trends of gene changes at the transcription level may not be fully consistent with the trends at the protein level, the potential mechanism needs to be further explored.

## Conclusion

In this study, 1) we analyzed the gene expression profiles of MS human brain tissue and screened out 133 EP-DEGs. 2) We predicted the biological processes and pathways they participate in. 3) We also predicted 4 key extracellular proteins and 16 extracellular proteins that may interact with IL17A. 4) We validated the levels of IL17A, Del-1, and resolvinD1 in clinical samples, performed survival analysis on Del-1, and found that Del-1 may be related to the relapse and progression of RRMS. The purpose of this study is to provide bioinformatics and clinical evidence for the discovery of potential biomarkers of MS.

## Data Availability Statement

Publicly available datasets were analyzed in this study. This data can be found here: https://www.ncbi.nlm.nih.gov/geo/query/acc.cgi?acc=GSE5839, NCBI GEO GSE5839.

## Ethics Statement

The studies involving human participants were reviewed and approved by The Ethics Committee of First Affiliated Hospital of Harbin Medical University (No. 2019118). The patients/participants provided their written informed consent to participate in this study. Written informed consent was obtained from the individual(s) for the publication of any potentially identifiable images or data included in this article.

## Author Contributions

ML conceived the idea, searched relevant information, and performed all bioinformatics analysis. Then ML collected the CSF of RRMS patients and control group patients, detected the levels of extracellular proteins by ELISA and followed up the patients. JS, FJ, and ZT helped ML to collect the CSF, and XF, CX, YW, BH, and HW helped ML to record and arrange the clinical data. Finally, ML drafted and finalized the article. DZ has been committed to studying the molecular immunomodulation effects in neuro-autoimmune diseases. She proposed the point that we should search for more detected biomarkers in body fluids of MS patients to improve the diagnosis and prognosis of MS. DZ reviewed and revised the manuscript many times. GL contributed to this manuscript by setting follow-up schedules, providing suggestions to reviewers’ comments, and modifying the manuscript. HC, PY, and YX reviewed the manuscript and provided many comments. All authors agree to be accountable for the content of the work. All authors contributed to the article and approved the submitted version.

## Funding

This work was supported by the National Natural Science Foundation of China (No. 81873773) and the Foundation of the First Affiliated Hospital of Harbin Medical University, China (Grant No. 2019M18).

## Conflict of Interest

The authors declare that the research was conducted in the absence of any commercial or financial relationships that could be construed as a potential conflict of interest.

## Publisher’s Note

All claims expressed in this article are solely those of the authors and do not necessarily represent those of their affiliated organizations, or those of the publisher, the editors and the reviewers. Any product that may be evaluated in this article, or claim that may be made by its manufacturer, is not guaranteed or endorsed by the publisher.
