# Supplementary figures and images for: Identification and Clinical Validation of Key Extracellular Proteins as the Potential Biomarkers in Relapsing-Remitting Multiple Sclerosis
Source: Front Immunol. 2021 Dec 7;12:753929. doi: 10.3389/fimmu.2021.753929 (PMC8688859; doi:10.3389/fimmu.2021.753929)

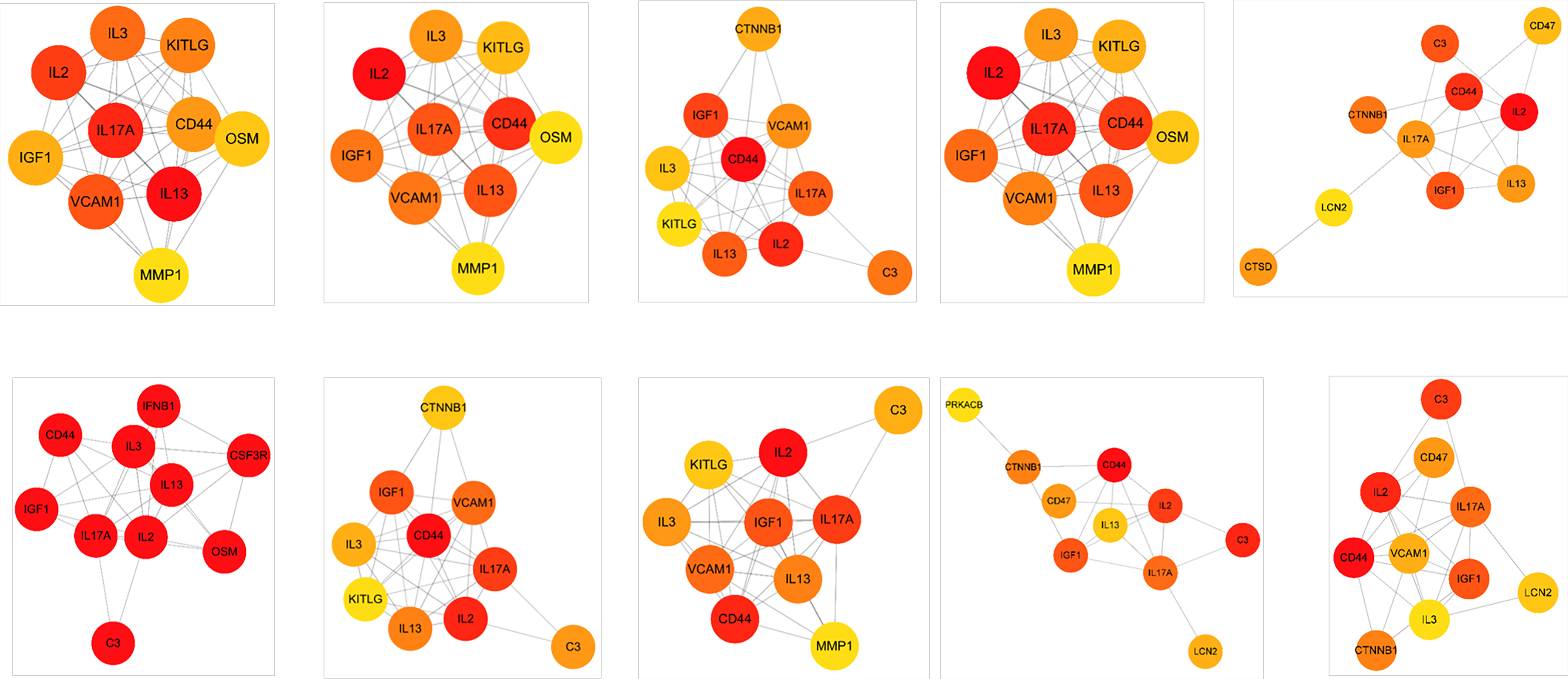

Supplement: Supplementary file 3 [file Image_2.tif]
